# A multi-layered governance framework for incorporating social science insights into adapting to the health impacts of climate change

**DOI:** 10.3402/gha.v6i0.21820

**Published:** 2013-09-10

**Authors:** Kathryn J. Bowen, Kristie Ebi, Sharon Friel, Anthony J. McMichael

**Affiliations:** 1 National Centre for Epidemiology and Population Health, Australian National University, Canberra ACT, Australia; 2 Department of Resource Management and Geography, University of Melbourne, Parkville, VIC, Australia; 3 ClimAdapt, LLC, Los Altos, CA, USA

**Keywords:** climate change adaptation, global health, multi-sectoral, social networks, governance, social sciences, methods

## Abstract

**Background:**

Addressing climate change and its associated effects is a multi-dimensional and ongoing challenge. This includes recognizing that climate change will affect the health and wellbeing of all populations over short and longer terms, albeit in varied ways and intensities. That recognition has drawn attention to the need to take adaptive actions to lessen adverse impacts over the next few decades from unavoidable climate change, particularly in developing country settings. A range of sectors is responsible for appropriate adaptive policies and measures to address the health risks of climate change, including health services, water and sanitation, trade, agriculture, disaster management, and development.

**Objectives:**

To broaden the framing of governance and decision-making processes by using innovative methods and assessments to illustrate the multi-sectoral nature of health-related adaptation to climate change. This is a shift from sector-specific to multi-level systems encompassing sectors and actors, across temporal and spatial scales.

**Design:**

A review and synthesis of the current knowledge in the areas of health and climate change adaptation governance and decision-making processes.

**Results:**

A novel framework is presented that incorporates social science insights into the formulation and implementation of adaptation activities and policies to lessen the health risks posed by climate change.

**Conclusion:**

Clarification of the roles that different sectors, organizations, and individuals occupy in relation to the development of health-related adaptation strategies will facilitate the inclusion of health and wellbeing within multi-sector adaptation policies, thereby strengthening the overall set of responses to minimize the adverse health effects of climate change.

Climate change may have serious and potentially catastrophic impacts over the longer term (1), depending on the choices that human populations and their governments make in the next 5–10 years. In the near term, climate will continue to change for at least several decades, irrespective of mitigation actions taken now. Effective and efficient adaptation may mean that even rapid and extensive climate change could be managed, at least temporarily, depending on the rate, magnitude, and extent of climate change.

Adaptation activities are important for protecting human health, as climate change poses many direct and indirect effects on health (2). Impaired food yields and lack of potable water, an increase in the occurrence of extreme weather events, as well as increased heat exposure, and the wider spread of vector-borne diseases present substantial physical and mental health challenges (3, 4).

Barriers to climate change action (in the public health field but also more broadly) have been identified as including the lack of financial incentives for research and development of new technologies, and organizations and individuals with vested interests supporting current development trajectories (5). Hence, an understanding of how decisions are being made to adapt, as well as who is (and is not) involved in making these decisions, is needed to improve and hasten our adaptation (and mitigation) efforts, particularly in relation to health.

Multi-level systems and cross-scale networks that link organizations and individuals are considered crucial for climate change adaptation (6, 7). This is particularly pertinent for adaptation activities to protect health because health is affected by many sectors that lie outside the direct purview of the sector itself – sectors such as water and sanitation, education, trade, agriculture, tourism, disaster management, development, and housing. For example, extreme weather events such as floods can have both direct health impacts (deaths caused by drowning) and indirect impacts (loss of agricultural productivity and a consequential increased rate of malnutrition; increase in diarrheal disease due to contaminated water). In this example, consideration of health becomes important for the agriculture, water and disaster management sectors, with implications for the development of more cross-sectoral adaptation activities to address the health risks of the exposure and to develop measures to manage the risks. Important here, too, is the need to understand the context-specific nature of climate change and its health effects, particularly for Indigenous and other communities who have a powerful attachment to a place (8).

Decision making in the context of climate change adaptation is complicated and challenging given the necessary involvement of multiple sectors and scales, including the increasing activity of actors ‘beyond the state’ such as non-government and private organizations. In addition, successful governance of adaptation to climate change also depends on appropriate, supportive, and enabling institutional structures (9–12).

An analysis of governance systems indicates how health adaptation strategies can be developed in ways that enable their incorporation into a broader-based systems approach – as would be needed in many inter-sectoral strategies. A greater understanding of decision-making processes and associated actors and organizations that yield power and influence will enhance the leveraging of policy access points. Such knowledge also enables realignment of adaptation activities to appropriately focus on individuals and populations whose health is most at risk from climate change.

Clarifying how adaptation decisions are being made and who is involved in this process – given the recognition that this involvement needs to be multi-level and cross-scale – requires the identification of a clear framework of governance components within which to make an assessment. This article presents a novel approach to define such a framework by combining an analysis of climate change adaptation, global health, and governance. This article synthesizes the current knowledge in these areas, with a particular focus on multi-level systems, cross-scale networks and institutional structures.

## Climate change, health, and links with other sectors

The links between climate change and human health have been made clear in recent years (2, 13, 14), showing that climate change has consequences beyond the environmental and economic spheres. In addition to the direct health and survival consequences of extreme weather events, many of the health risks associated with climate change arise less directly via pathways relevant to the agriculture, water and sanitation, transport, disaster management, planning and health sectors.

Climate-related health effects are, and will be, inequitably distributed, with developing countries and socially disadvantaged groups generally facing more severe outcomes (13, 15, 16) due to their underlying levels of disease risk and occurrence, lack of infrastructure and poor living conditions, weak economies, insufficient emergency management, and often poor governance processes. Governance is an important element of the broad societal level determinants of health – that is, the factors that underlie the health status of communities, countries, and regions. Via diverse paths, governance influences the way that the factors that affect states of health are created and distributed among and between populations (17).

### Governance as a determinant of adaptive capacity

Governance, as the prime medium for taking social decisions and actions, is a determinant of adaptive capacity (18–20), the strengthening of which can reduce vulnerability to the health effects of climate change (13). Adaptation actions have two major categories: development of strategies, policies; and measures and implementation (6). When done well, both categories use a suite of governance-related functions, including clear mandates, inclusive and effective decision making and response to community-identified strengths, and material and non-material resource requirements. An understanding of governance structures and decision-making processes helps to articulate the pathways that lead to policy development and implementation within and between different sectors (19–21).

Although a multi-sectoral governance approach is necessary for effective and efficient climate change adaptation, this is not generally the normal operating approach of governments or, indeed, of many non-government organizations. Challenges are evident when working beyond the usual silos – from organizational differences such as structures and processes, to individual differences, such as knowledge, willingness, and an understanding of ‘the bigger picture’. These differences are already present within organizations, so the capacity for organizations to work beyond their given portfolios magnifies these common problems.

The concept of ‘earth system governance’ is broader than states and governments, describing all levels of decision making by public and private actors, including NGOs, private corporations, UN agencies, and individual experts (Biermann, 2007). Given the importance of developing and supporting adaptive capacity in health and other sectors via understanding and strengthening decision making and governance structures, it is vital to understand the links (or lack thereof) between and within relevant sectors. In addition, identifying ‘catalysts’ for adaptation policy and activity can enhance advocacy efforts by encouraging the factors that appear to be working and reducing (where possible) those factors that inhibit adaptation action.

Consideration of the articulation of equity, influence, and power should illuminate how decisions are made and their policy context (21). Therefore, three specific concepts from the earth system governance framework are of relevance to this study – ‘agency’, ‘architecture’, and ‘adaptiveness’ (22). Agency refers to the actors, formal and informal, government and non-government that have governance functions. Architecture explores decision-making processes and governance beyond single (environmental) institutions (23). Adaptiveness describes the capacity for change (in this case policy changes) within the system of governance itself (as well as subsequently referring to the governance of adaptation actions in response to social–ecological change). All three concepts highlight the importance of looking beyond formal single-layered decision-making structures and processes.

The following section outlines a theoretical framework with which to evaluate these concepts of interest within the context of climate change and health adaptation decision making.

### Systems of governance and the ‘goodness of fit’

The often misaligned connection between institutions, agencies, and organizations and the ecosystems that they are designed to manage or govern is referred to as the ‘problem of fit’ (24). The problem (or goodness) of fit is an idea that has been used predominantly in the context of ecosystem-based management, and can be seen as the inverse of an ‘enabling environment’. This concept of ‘fit’ can also usefully be applied to public health governance to understand the important and influential individuals and organizations that are involved in developing adaptation strategies relevant to the health risks of climate change, as well as the broader policy context.

Decision making for health often involves not just the health sector but also agriculture, water, disaster management, and others. In assessing health governance, understanding which organizations and individuals are perceived as key agents in the decision-making process can guide approaches to leverage those deemed to be influential or powerful. Gaps or ‘misfits’ in institutional arrangements, which fail to fit the system of governance, can also be identified, and an understanding and reduction of these gaps can lead to an improvement of governance. This holistic perspective and analysis of relevant sectors and institutions provides a (necessarily) fuller understanding of the system and possible institutional gaps (24, 25).

**Figure 1:**
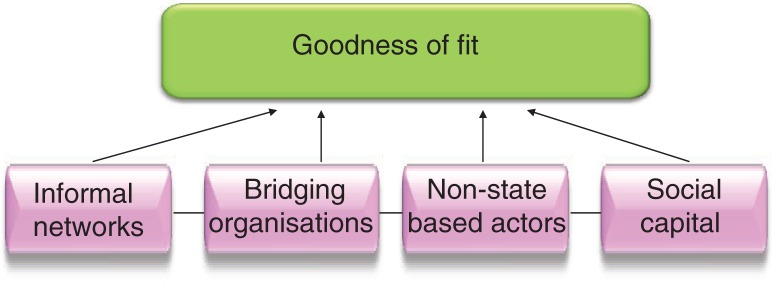
Four connected components of the governance and decision-making context in relation to CCA and health, which combine to determine the goodness of ‘fit’.

Four governance elements are postulated here to create the governance environment for health and climate change adaptation (Fig. 1): (1) social capital; (2) non-state-based actors; (3) informal networks, and (4) bridging organizations. These elements combine to influence the ‘fit’ of the governance context. These components must interlink and need to be considered as a whole for a valid assessment of ‘fit’.

A multi-layered approach is adopted here to understand the ‘network and policy map’ – that is, the actual decision-making actors, their roles, and their level of influence. By studying the whole inter-organizational network beyond just organizational levels of analysis, we can understand the way collective outcomes may be achieved (26).

These four connected components that interweave to produce the goodness of fit for decision making in relation to climate change adaptation and global health are described in more detail below.

#### Social networks and social capital

Social networks play a key role in adaptive governance, as these often self-organize and pool experiences and knowledge to shape change (27). Social capital is crucial to the operationalizing of adaptive governance (27). The links between social capital, health (in particular mental health), and climate change have begun to be explored [see review in Berry et al. (4)], but the links between these factors and adaptive governance have not yet been thoroughly examined. The importance of social networks in enhancing communities’ adaptive responses to environmental change and in supporting governance mechanisms has been identified (28, 29). Understanding such social networks also requires an appreciation of the influence of social capital.

Social capital can be viewed as the capacity of a population to work harmoniously as a self-organizing unit, in which many individuals co-operate, but in which no single person, or even group, controls all activities. Instead of ‘top-down’ control, groups function because individuals and groups learn and acquire norms and customs from their parents, families, schools, and society that inform and influence behavior—whether or not this is a positive force depends on the circumstances. Four central aspects of social capital are relations of trust; reciprocity and exchanges; shared rules, norms and sanctions; connectedness, networks and groups (30). These components display a substantial crossover with the components of adaptive governance. Furthermore, it has been argued that community-based adaptation has social capital at its core (31).

Ties within defined groups, such as friendship and kinship, are often known as ‘bonding’ social capital. These bonds can be a vital compensation for low income and socially excluded groups, particularly where social security provision is weak. In contrast, economic and other ties to wider groups are usually based on weaker bonds of trust and reciprocity. Such ‘networking’ or ‘bridging’ social capital may rely on legal and formal institutions. It is important to recognize that not all social networks are created equal. In particular, networks composed only of bonding links, which foster group homophily (the tendency of individuals to bond with similar others) and constrain social norms, can reduce resilience (and hence adaptive capacity). This is in contrast to networks that are composed of bridging links when the diverse resources that are available to communities strengthen their ability to cope and adapt to change (32). In general, a good mix of bonding and bridging networks will lead to greater resilience and adaptability (29).

Social capital is integrally linked to both the health of the natural environment and the human population. Securing livelihoods and maintaining wellbeing (at least partly) results from levels of social capital that enhance shared access to resources (33). Some have argued that development assistance has paid too little attention to how social (and human) capital affects environmental outcomes (30). In terms of social capital and health, communities that present higher levels of social cohesion are more effective at accessing services and amenities (34). In addition, social capital may be related to the incidence of violent crime, as shown by research conducted in the United States (35).

#### Actors beyond the state

The importance of understanding social networks in a more holistic and systems-based approach is emphasized by the growing literature on ‘actors beyond the state’ that identifies the increasing relevance of non-state actors in influencing environmental governance processes (36) and more general governance processes (37). In addition to the multi-sectoral and multi-scale nature of adapting to the health effects of climate change, the past decade has seen the strong emergence of actors that lie outside the traditional state-based decision-making structures and processes. Donor countries, development banks, and the United Nations are increasingly focusing attention on enhancing financial and technical support for adaptation initiatives – including many that, although not explicitly directed at human health, have relevance for health. This can be seen by the influx of adaptation activities that are funded by bilateral institutions (e.g. Australian Agency for International Development, Danish International Development Agency) as well as multilateral institutions (e.g. European Commission, the World Bank) and international non-government organizations (e.g. Red Cross, Oxfam).

Building trust and cooperation between actors inside and outside the state structures, such as civil society, may yield co-benefits in the context of adaptation (38). One benefit is that synergistic social capital and inclusive decision-making institutions promote the sustainability and legitimacy of adaptation strategies. The second is that adaptation processes that are bottom-up and based on social capital can shift the perception of climate change from a too-distant global problem to a more tangible local problem. Although this sounds sensible in theory, the current chaotic influx of organizations becoming involved in climate change adaptation presents challenges for the development of adaptation strategies that align with these principles.

#### Informal networks

Formal and informal networks are important to consider when evaluating governance structures and decision-making processes. Informal networks, or ‘shadow networks’, are important for the development of new ideas and creativity, and for the flow of information (7) outside more typical formal network structures. It may well be that this ‘mess of interactions’ is as important for long-term capacity to adapt to global environmental change as much as the formal organizational structures (39). The development and effectiveness of shadow networks is highly dependent on leadership (7). Shadow networks are the focus of research in governing social–ecological systems and have not explicitly included the health sector.

#### Bridging organizations

Another important component of system-wide governance is bridging organizations that link groups, networks, and organizations across levels and create the right links between individuals, issues, and timing (27, 40). The emergence of bridging organizations seems to lower the costs of collaboration by accessing and consolidating various avenues of knowledge and interest to respond to social–ecological change (27). Although socio–ecological change has been the focus of research on bridging organizations, an understanding of bridging organizations is also useful for the health arena. It is anticipated that bridging organizations play an important role in the context of climate change adaptation because although policy development is generally conducted at a central government level, adaptation activities occur on a local scale. Organizations that act as links between these different scales may therefore increase the likelihood of inclusive and effective decision-making processes for adaptation policy and activity.

A better understanding of these four components of adaptive governance – social networks and capital, actors beyond the state, informal networks, and bridging organizations – and how their makeup influences decision-making processes, will enhance the portfolio of tools available to increase resilience. The field of climate change adaptation and global health brings with it new challenges which necessitate approaches that consider these broader elements of governance.

## Discussion and conclusion

This article presents a framework that can be used to assess governance structures and decision-making processes, with specific relevance to climate change adaptation for health. An understanding of the various elements of the goodness of fit presented here allows a fuller appreciation of the governance environment, thereby ultimately increasing our potential to strengthen these elements. This framework is being tested in an evaluation of decision-making processes in the development of adaptation options relevant to the health sector in research underway in Cambodia, Vietnam, and Fiji. Social network research will be used as part of a broader policy and social analysis to describe and evaluate the relationships and bonds between sectors and actors.

Understanding governance systems for adaptation is vital given current and future substantial monetary investments in adaptation activities; that is, identifying who is involved in making policy and practical decisions relating to climate change and health, and the enabling and hindering factors for different contexts. There is a need to depart from a business as usual ‘silo-ed’ approach to health, to one that includes evaluating and understanding decision-making processes and links between health and other sectors that are not always considered within the health context, but are fundamental to health and adaptation.

Importantly, climate change will exacerbate current health burdens, many of which are the subject of aspirational goals to reduce global rates of disease, such as in the Millennium Development Goals. Despite the grave predictions that are given for climate change inaction, and knowledge of its health effects, it is clear that policymakers are not moving quickly enough. Events such as the disappointing lack of outcomes from the Rio+20 conference, the global financial slowdown taking precedence over other public policy areas (including climate change), and the distracting nature of the debate surrounding climate change attribution that prohibits climate change action, all contribute to the sombre reality regarding a lacklustre climate change mitigation response. However, there is substantial potential for adaptation funding, policy, and action to redress the current global health imbalance. The global health community (research, policy, and practice), working with relevant sectors and agencies, has an obligation to seize and capitalize on this opportunity to improve the health and wellbeing of vulnerable populations and communities.
